# Predictors for aki in a cardiac surgery population undergoing cardio-pulmonary bypass

**DOI:** 10.1186/2197-425X-3-S1-A635

**Published:** 2015-10-01

**Authors:** F Ramakers, Q Swennen, V Pennemans, J Penders, M Vander Laenen, W Boer

**Affiliations:** Intensive Care and Anaesthesiology, Ziekenhuis Oost Limburg, Genk, Belgium; Biomedical Research Institute, UHasselt, Diepenbeek, Belgium

## Introduction

In cardiac surgery involving cardio-pulmonary bypass, AKI is a relatively common diagnosis. In recent years a number of new biomarkers have been undergoing validation in this clinical setting. It is thought that earlier detection of AKI will offer opportunities for new therapeutic interventions

## Objectives

Analysis of a database was performed to define baseline patient and biomarker characteristics in patients developing AKI in a population of cardiac surgery patients undergoing cardiopulmonary bypass as part of the procedure.

## Methods

In total, 259 patients were enrolled, after the exclusion of patients with severe pre-existing renal insufficiency (defined as a eGFR< 15 ml/min). As part of a broader study, urine and blood samples were obtained immediately before initiation of cardio-pulmonary bypass. Patients were subsequently retrospectively divided into 2 groups, AKI (n=84) and non-AKI (n=175). This subdivision was based on based on the AKIN criteria i.e. increase in s-Creat ≥ 0.3 mg/dl or ≥ 50% compared to baseline within 48 h or reduction in urine output < 0.5 ml/kg/h for more than 6 h. Statistical analysis of all characteristics before arrival on the ICU was performed.

## Results

AKI patients (32% of total) were older (70 yrs (SD = 9) vs 67 (11), p =0.043) with higher BMI´s (27.7 (4.8) vs.26.7 (4.3), p = 0.036). As to be expected baseline eGFR (CKD-EPI, in ml/min) was lower in the AKI-group (69.49 (20.30) vs. 76.45 (15.01), p = 0.024). Both baseline urinary-NGAL (µg/l)(1211 (2172) vs.749 (946), p = 0.020) and serum-cystatin C (in mg/L)(0.98 (0.39) vs 0.86 (0.36), p = 0.0175) were statistically higher in the AKI group and CPB time (in minutes) was significantly longer: 163 (63) vs 121 (51), p < 0.0001.

## Conclusions

Urinary-NGAL and serum-Cystatin most likely reflect pre-existing kidney dysfunction (like eGFR). Length of cardiopulmonary bypass time is a significant factor for development of AKI, which is amenable to improvement.
